# Optics of spatiotemporal optical vortices for atto- and nano-photonics

**DOI:** 10.1515/nanoph-2024-0544

**Published:** 2025-01-06

**Authors:** Miguel A. Porras

**Affiliations:** Grupo de Sistemas Complejos, ETSIME, Universidad Politécnica de Madrid, Rios Rosas 21, 28003 Madrid, Spain

**Keywords:** structured light, orbital angular momentum, spatiotemporal optical vortices

## Abstract

Understanding the intricate properties of spatiotemporal optical vortices (STOVs) is crucial for their growing number of applications, particularly where they drive light–matter interactions that can create up-converted STOV-like structures at attosecond and nanometer scales. We derive closed-form expressions for the propagation of STOVs, and spatiotemporal (ST) tilted Hermite-lobed (THL) pulses forming STOVs, through cascaded optical systems represented by *ABCD* matrices such as free space propagation, lenses, mirrors, similar in simplicity as those in Gaussian beam optics. STOVs and ST THL pulses are found to be spatial and temporal Fourier transform pairs at the same time, so that a STOV is a spatiospectral (SS) THL pulse in SS domain and a ST THL pulse is a SS vortex. This duality allow us to find expressions for the transformation of their spatiospectra through arbitrary optics, which is particularly important at the attosecond and nanometer scales, where ST characterization techniques are limited.

## 1 Introduction

Spatiotemporal optical vortices (STOVs), spatiotemporal fields with a transverse line phase singularity, were theoretically described about two decades ago [1], [2], and first observed in a nonlinear optics experiment as a side-effect of the arrest of collapse in filamentation [3]. As a fundamentally linear wave object, they were subsequently generated by purely linear means, typically with a 4*f* pulse shaper with a tilted *π*-step phase plate, a spiral phase plate [4], or a spatial light modulator [5], placed at the Fourier plane. Other more compact devices have afterwards been proposed or realized [6], [7], [8], see also [9] for a review. Last years, STOVs are once again being generated by nonlinear means from visible and near infrared STOVs that drive perturvative and nonperturbative, strong-field light–matter interactions, and up-convert their structure to second-order harmonic [10], [11], sum-frequency STOVs in ultraviolet [12], and third-order harmonic STOVs [13]. Theoretical studies of high-order harmonic and attosecond STOVs have been reported in [14], [15], and they have been observed recently [16], giving birth to STOVs at the attosecond and nanometer scales.

The precise knowledge of the spatiotemporal structure of STOVs, its change on propagation or focusing systems is crucial for the precise control of the driving field and its interaction with matter. This knowledge is also required for the interpretation of the presumably STOV-like structures observed in the generated harmonics or attosecond pulses. After preliminary numerical studies [17], [18], [19], the free space propagation of STOVs, and of their dual fields, or spatiotemporal tilted Hermite-lobed (THL) pulses, were studied analytically in [20], and focusing of ST THL pulses producing STOVs in [21]. Focusing of STOVs themselves has only been inspected numerically [17], [18], [22].

We develop a theory of the optics of STOVs similar in simplicity to the theory of Gaussian beam propagation through optical systems represented by *ABCD* matrices. Material dispersion in the elements is assumed to be small, as intended in the experiments. Since STOVs are not continuous beams we develop alternate formulations in spatioemporal (ST) and spatiospectral (SS) domains that would describe the observations with spatial and temporal resolution, and the observations of spatially resolved spectra. The ST description is the most common with visible or near infrared STOVs as they are usually long pulses of several tens of femtoseconds where characterization techniques are standard. Interestingly, second-order harmonic STOVs are still observed in ST domain [4], [10], third-order harmonic STOVs are observed in both ST and SS domains [13], but the high-order harmonics in [16] can only be characterized experimentally in SS domain given the limitations of ST characterization techniques at attosecond and nanometer scales.

**Figure 1:**
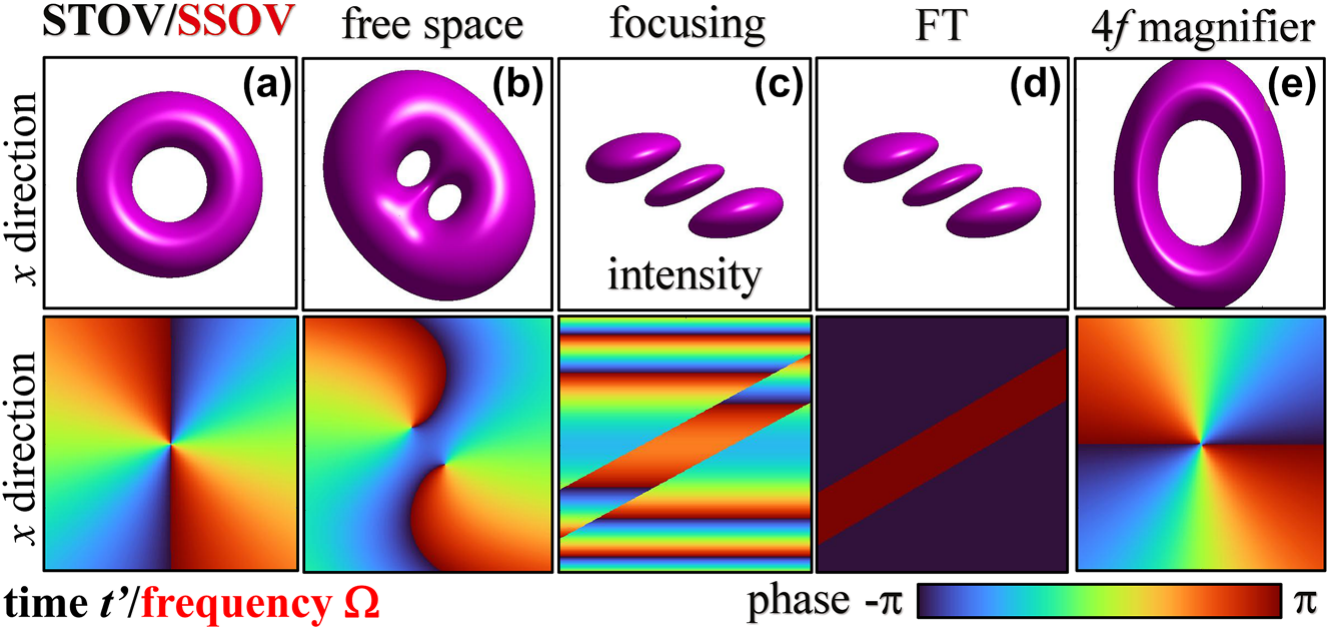
Transformation of STOVs and SSOVs through various optical systems. The TC is +2 in this example. (a) STOV or SSOV, and their (b) free space propagation, (c) focusing, (d) Fourier transform, (e) 4*f *system magnification. First row: Iso-intensity surface. Second row: phase.

**Figure 2:**
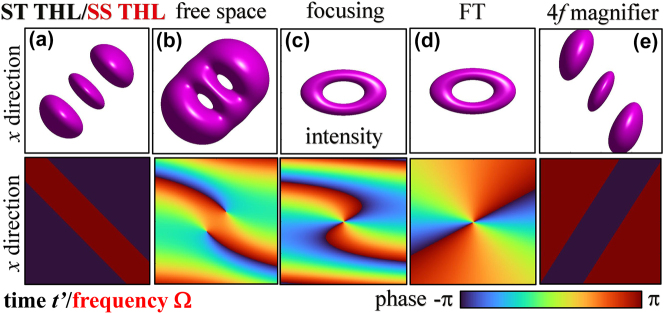
Transformation of ST THL and SS THL pulses through various optical systems. The Hermite polynomial order is 2 in this example. (a) ST THL or SS THL, and their (b) free space propagation, (c) focusing, (d) Fourier transform, (e) 4*f *system magnification. First row: Iso-intensity surface. Second row: phase.

A complete picture of the ST propagation of STOVs, as that seen in Figure 1(a) with the temporal abscissa, needs the supplementary ST THL pulse seen in Figure 2(a), also with the temporal abscissa. STOVs and ST THL pulses are the two complementary output pulses from a standard 4*f* pulse shaper when the tilted *π*-step phase plate or the spiral phase plate is placed at the common focus, and constitute a spatial Fourier transform pair. The spatiospectrum of STOVs have been repeatedly observed or numerically evaluated [13], [16], but only calculated for unit topological charge (TC) [14]. It turns out that the temporal Fourier transform of a STOV of arbitrary TC is the SS THL pulse, also in Figure 2(a) with the frequency abscissa, and the temporal Fourier transform of the ST THL pulse is the SS optical vortex (SSOV), seen Figure 1(a) with the frequency abscissa. Thus, a complete picture of propagation in SS domain requires the same pair of pulses. In addition, the symmetry of the propagation equations for paraxial and quasi-monochromatic pulses in ST and SS domains implies that the propagation of STOVs or ST THL pulses is identical to the propagation of SSOVs or SS THL pulses. This duality has implications such as, for example, that one may observe the spatially resolved spectrum of a STOV created by a 4*f* pulse shaper without any spectrometer by simply replacing the tilted *π*-step phase plate with the spiral phase plate, or vice versa for a ST THL pulse, and the same after propagation though any optical elements. For the extreme ultraviolet harmonic or attosecond STOVs generated at a focus, one would observe their ST structure at that focus with a spatially resolved spectrometer at the far field. It was in fact the numerical observation of this duality that led to the interpretation of the high harmonics in [16] as STOVs.

We illustrate the theory with focusing and propagation up to the far field as the most common *ABCD* system in experiments with STOVs/ST THL pulses, providing complete, closed-form expressions for STOV and ST THL pulse focusing in ST and SS domains, and unveil unexpected features at the far field that should be taken into account in experiments.

Also, once the debate on the transverse orbital angular momentum (OAM) of STOVs has been closed [23], we find that *ABCD* systems such as a lens and a Fourier transforming system, or image-forming systems such a single lens or a 4*f* magnifier, can manipulate the transverse OAM as desired, imparting or removing it, even changing its direction, which will be of interest for the future applications of the transverse OAM.

## 2 Propagation of STOVs and ST THL pulses through optical systems

Under paraxial and quasi-monochromatic conditions, the ST envelope of an optical field in non-dispersive media is ruled out by the Schrödinger equation *∂*_*z*_ *ψ* = (*i*/2*k*_0_)Δ_⊥_ *ψ*, where *k*_0_ = *ω*_0_/*c* is the propagation constant, *ω*_0_ is the carrier frequency, *c* the velocity of light, and Δ_⊥_ the transverse Laplacian to the propagation direction *z*. In absence of dispersion, the local time *t*′ = *t* − *z*/*c* does not appear explicitly in the Schrödinger equation since the second-order derivative in the local time vanishes. Ideally, STOVs and ST THL pulses have separable fields of the form *ψ*_0_(*x*, *y*, *t*′) = *X*_0_(*x*, *t*′)*Y*_0_(*y*). Separability allows us to use the 1D version of Schrödinger equation $\partial_{z}X=\left(i/2k_{0}\right)\partial_{x}^{2}X$ for the propagation of *X*_0_ and omit from now the independent propagation of *Y*_0_. The solution of the 1D Schrödinger equation can then be written in terms of the 1D Fresnel diffraction integral as

(1) $$X_{1}\left(x,t^{\prime }\right)=\sqrt{\frac{k_{0}}{2i\pi z}}\int dx^{\prime }X_{0}\left(x^{\prime },t^{\prime }\right)e^{\frac{ik_{0}}{2z}{\left(x-x^{\prime }\right)}^{2}},$$

for propagation a distance *z*, where *t*′ appears only as a parameter in the initial pulse. These fields may additionally experience propagation through a series of aligned optical systems that can be represented by an *ABCD* matrix and that do not introduce significant material dispersion. If these systems do not break the separability in *x* and *y*, the propagation of *X*_0_(*x*, *t*′) can be written in terms of the generalized Fresnel diffraction integral as [24]

(2) $$X_{1}\left(x,t^{\prime }\right)=\sqrt{\frac{k_{0}}{2i\pi B}}\int dx^{\prime }X_{0}\left(x^{\prime },t^{\prime }\right)e^{\frac{ik_{0}}{2B}\left(Dx^{2}-2xx^{\prime }+Ax^{\prime 2}\right)}.$$

### 2.1 Propagation of STOVs

An elliptical STOV with transverse OAM along the *y* direction is described by [25]

(3) $$X_{0}\left(x,t^{\prime }\right)={\left(\frac{t^{\prime }}{t_{0}}\mp i\frac{x}{x_{0}}\right)}^{n}e^{-\frac{x^{2}}{x_{0}^{2}}}e^{-\frac{t^{\prime 2}}{t_{0}^{2}}},$$

whose intensity and phase are illustrated in Figure 1(a). The parameters *t*_0_ and *x*_0_ determine the STOV duration and transversal size, and the natural number *n* > 0 determines the topological charge of the vortex as ±*n* for the ∓ signs in Eq. (3). The opposite signs compared to [25] are due to the fact that the negative TC in the *t*′ − *x* plane is actually positive TC in *z* − *x* plane.

With the STOV in Eq. (3), long but straightforward algebra including adequate changes of variables allow us to identify the integral in Eq. (2) with integral 3.462.4 in [26]. Once the changes are reverted, we obtain the closed-form expression for the STOV propagation in an arbitrary, but non-dispersive, *ABCD* system:

(4) $$\begin{array}{ll} X_{1}\left(x,t^{\prime }\right) & =\sqrt{\frac{1}{A\;+\;\;B/q_{0}}}e^{\frac{ik_{0}x^{2}}{2q_{1}}}e^{-\frac{t^{\prime 2}}{t_{0}^{2}}}{\left(\frac{B/q_{0}}{A\;+\;\;B/q_{0}}\right)}^{\frac{n}{2}}\\ & \;\times \frac{1}{2^{n}}H_{n}\;\left[\;\sqrt{\frac{A\;+\;\;B/q_{0}}{B/q_{0}}}\;\left(\frac{t^{\prime }}{t_{0}}\mp i\frac{x}{x_{0}}\frac{1}{A\;+\;\;B/q_{0}}\right)\;\right]. \end{array}$$

Here, *H*_*n*_(⋅) is the Hermite polynomial of order *n*, and *q*_0_ and *q*_1_ are the standard complex beam parameters of the input and output Gaussian beam enveloping the STOV, related by the well-known *ABCD* law in standard Gaussian beam optics:

(5) $$\frac{1}{q_{0}}=\frac{2i}{k_{0}x_{0}^{2}},\;\frac{1}{q_{1}}=\frac{C+D/q_{0}}{A+B/q_{0}}.$$

Equation (4) supplemented by Eq. (5) is our first main result. The validity of the *ABCD* law for the particular case of focusing a ST THL pulse to a STOV of unit TC has been noticed in [27]. A few common optical experimental setups are considered below. We should be warned that the intrinsic transverse OAM of STOVs is conserved in free space propagation [22], but is not generally conserved upon passage through a general *ABCD* system containing lenses and mirrors [21]. Also, TC changes occur even in free space propagation [22], [25]. Depending on the particular *ABCD* system, TC changes may or may not be accompanied by changes in the transverse OAM. For aligned systems and STOVs/ST THL pulses, we limit our considerations to the intrinsic transverse OAM since the total OAM about a *y* axis across *x* = 0 vanishes and the extrinsic is opposite to the intrinsic [22]. For brevity, we will omit the word intrinsic.

For *free space propagation,* Eq. (4) with (*A*, *B*, *C*, *D*) = (1, *z*, 1, 0) reduces to

(6) $$\begin{array}{ll} X_{1}\left(x,t^{\prime }\right) & =\sqrt{\frac{-iz_{R}}{q_{1}}}e^{\frac{ik_{0}x^{2}}{2q_{1}}}e^{-\frac{t^{\prime 2}}{t_{0}^{2}}}{\left(\frac{z}{q_{1}}\right)}^{\frac{n}{2}}\\ & \;\times \frac{1}{2^{n}}H_{n}\left[\sqrt{\frac{q_{1}}{z}}\left(\frac{t^{\prime }}{t_{0}}\mp \frac{x}{x_{0}}\frac{z_{R}}{q_{1}}\right)\right], \end{array}$$

with $z_{R}=k_{0}x_{0}^{2}/2$ and *q*_1_ = *z* − *iz*_*R*_, which was previously written in [20]. The ±*n*-charged vortex splits into *n* ± 1-charged vortices, as seen in Figure 1(b), but neither the total TC nor the transverse OAM experience any change.

Surprisingly, *focusing* of a STOV has only been studied by numerical means [17], [18], [22]. Equation (4) with the matrix (*A*, *B*, *C*, *D*) = (1 − *z*/*f*, *z*, − 1/*f*, 1) for focusing with focal length *f* and propagation a distance *z* beyond yields the closed-form expression

(7) $$\begin{array}{ll} X_{1}\left(x,t^{\prime }\right) & =\sqrt{\frac{q}{q_{1}}}e^{\frac{ik_{0}x^{2}}{2q_{1}}}e^{-\frac{t^{\prime 2}}{t_{0}^{2}}}{\left(\frac{iqz}{q_{1}z_{R}}\right)}^{\frac{n}{2}}\\ & \;\times \frac{1}{2^{n}}H_{n}\left[\sqrt{\frac{q_{1}z_{R}}{iqz}}\left(\frac{t^{\prime }}{t_{0}}\mp i\frac{x}{x_{0}}\frac{q}{q_{1}}\right)\right], \end{array}$$

where we have introduced the complex beam parameter 1/*q* = −1/*f* + 1/*q*_0_ immediately after the lens to compact the expression, and *q*_1_ = *q* + *z*. Equation (7) describes the splitting of the *n*-charged vortex into *n* vortices with identical TCs equal to +1 or −1 upon focusing, their disappearance at the focal plane and reappearance with opposite sign of the TC. At the focal plane, the *n* vortices of TCs ±1 degenerate in *n* tilted lines of zero intensity between the *n* + 1 lobes of the ST THL pulse

(8) $$X_{1}\left(x,t^{\prime }\right)=\sqrt{\frac{-ix_{0}}{x_{0,f}}}e^{-\frac{x^{2}}{x_{0,f}^{2}}}e^{\frac{ik_{0}x^{2}}{2f}}e^{-\frac{t^{\prime 2}}{t_{0}^{2}}}\frac{1}{2^{n}}H_{n}\left(\frac{t^{\prime }}{t_{0}}\mp \frac{x}{x_{0,f}}\right),$$

where *x*_0,*f*_ = 2*f*/*k*_0_ *x*_0_ is the focal Gaussian width. The intensity and phase distributions of the focused ST THL pulse are seen in Figure 1(c). We note that the input STOV carries ±*nγ*/2*ω*_0_ transverse OAM per photon, with *γ*_0_ = *ct*_0_/*x*_0_ the ellipticity, that the lens does not add or remove transverse OAM to a wave with the symmetry of the elliptical STOV [21], and that the OAM is preserved during the free propagation beyond the lens. Thus, the reversal of the sign of the TC is not associated with any change of the transverse OAM.

Next we consider a *spatial Fourier transform system*. With the matrix (0, *f*, − 1/*f*, 0) for propagation from the back to the front focus of a lens, Eq. (4) yields

(9) $$X_{1}\left(x,t^{\prime }\right)=\sqrt{\frac{-ix_{0}}{x_{0,f}}}e^{-\frac{x^{2}}{x_{0,f}^{2}}}e^{-\frac{t^{\prime 2}}{t_{0}^{2}}}\frac{1}{2^{n}}H_{n}\left(\frac{t^{\prime }}{t_{0}}\mp \frac{x}{x_{0,f}}\right).$$

The only difference with Eq. (8) is the absence of the wave front curvature, as is clear from Figure 1(c) and (d). With a flat wave front, this ST THL pulse does not carry any transverse OAM [22]. Thus, the transverse OAM ±*nγ*_0_/*ω*_0_ of the STOV has been removed by the lens.

Particularly interesting is an *object-image system* as it manipulates the sign of the TC, and the amount and direction of the transverse OAM at will. With the general matrix of this system, (*M*, 0, *C*, 1/*M*) [24], the argument of the Hermite polynomial in Eq. (4) is singular, but this singularity is removed in the highest power term of the Hermite polynomial by the pre-factor ${\left[\left(B/q_{0}\right)/\left(A+B/q_{0}\right)\right]}^{n/2}$ , which in turn cancels all lower power terms. Also, the factor 1/2^*n*^ cancels the coefficient 2^*n*^ of the highest power term. Eq. (4) then reduces to

(10) $$X_{1}\left(x,t^{\prime }\right)=\sqrt{\frac{1}{M}}e^{\frac{ik_{0}x^{2}}{R_{1}}}e^{-\frac{x^{2}}{x_{1}^{2}}}e^{-\frac{t^{\prime 2}}{t_{0}^{2}}}{\left(\frac{t^{\prime }}{t_{0}}\mp i\mathrm{sgn}\left(M\right)\frac{x}{x_{1}}\right)}^{n},$$

where *x*_1_ = |*M*|*x*_0_, *R*_1_ = *M*/*C*, and sgn(*M*) is the sign of *M*. With *M* > 0, Eq. (10) is a new STOV of the same TC and transverse OAM of the same direction but different magnitude, ±*nγ*_1_/2*ω*_0_ as ellipticity has changed to *γ*_1_ = *γ*_0_/*M*. Two simple cases with *M* < 0 are the object-image with a single lens forming a real image, for which *M* = −*s*′/*s* is negative (*s* and *s*′ are the object and image distances) and *C* = −1/*f*, and a 4*f* magnifier, for which *M* = −*f*′/*f* (*f* and *f*′ the first and second lenses) and *C* = 0. Then Eq. (10) represents a new STOV of opposite TC, and transverse OAM in the opposite direction and different magnitude ∓*nγ*_1_/2*ω*_0_. The particular case of STOV at the output plane of the 4*f* magnifier is shown in Figure 1(e). Noteworthy, after removal of the transverse OAM by the first lens, the second lens imparts again transverse OAM to form the new STOV. In this respect, a recent and rather sophisticated experiment in Ref. [28] involving three synchronized lasers is claimed to constitute the only procedure to impart *spatiotemporally* transverse OAM. However, a single lens was described in detail to impart, also spatiotemporally, transverse OAM in [21], and simple optical systems such as a Fourier lens, an object-image system, or a 4*f* magnifier, can impart or remove it, even control its direction.

### 2.2 Propagation of spatiotemporal THL pulses

The dual field of a STOV from a 4*f* pulse shaper is the ST THL pulse

(11) $$X_{0}\left(x,t^{\prime }\right)=\frac{1}{2^{n}}H_{n}\left(\frac{t^{\prime }}{t_{0}}\pm \frac{x}{x_{0}}\right)e^{-\frac{x^{2}}{x_{0}^{2}}}e^{-\frac{t^{\prime 2}}{t_{0}^{2}}}.$$

Its intensity and phase are illustrated in Figure 2(a). The transformation through *ABCD* systems can be evaluated in a similar way as above from Fresnel diffraction integral in Eqs. (2) with (11), but using now integral 7.374.8 in [26]:

(12) $$\begin{array}{ll} X_{1}\left(x,t^{\prime }\right) & =\sqrt{\frac{1}{A\;+\;\;B/q_{0}}}e^{\frac{ik_{0}x^{2}}{2q_{1}}}e^{-\frac{t^{\prime 2}}{t_{0}^{2}}}{\left(\frac{A}{A\;+\;\;B/q_{0}}\right)}^{\frac{n}{2}}\\ & \;\times \frac{1}{2^{n}}H_{n}\;\left[\;\sqrt{\frac{A\;+\;\;B/q_{0}}{A}}\;\left(\frac{t^{\prime }}{t_{0}}\pm \frac{x}{x_{0}}\frac{1}{A\;+\;\;B/q_{0}}\right)\;\right], \end{array}$$

which is the second relevant result of this paper.

For *free space propagation,* Eq. (12) reduces to

(13) $$\begin{array}{ll} X_{1}\left(x,t^{\prime }\right) & =\sqrt{\frac{-iz_{R}}{q_{1}}}e^{\frac{ik_{0}^{2}}{2q_{1}}}e^{-\frac{t^{\prime 2}}{t_{0}^{2}}}{\left(\frac{-iz_{R}}{q_{1}}\right)}^{\frac{n}{2}}\\ & \;\times \frac{1}{2}H_{n}\left\{\sqrt{\frac{q_{1}}{-iz_{R}}}\left[\frac{t^{\prime }}{t_{0}}\pm \frac{x}{x_{0}}\left(\frac{-iz_{R}}{q_{1}}\right)\right]\right\} \end{array}$$

with *q*_1_ = *z* − *iz*_*R*_, which coincides with the result reported in [20]. The tilted lobes form *n* vortices of TC ±1 for *z* > 0, as in Figure 2(b), and ∓1 at *z* < 0 that only converge in a single vortex of TC ±*n* for *z* → +∞ and ∓*n* for *z* → −∞. This field does not carry any transverse OAM [20]. *Focusing* the ST THL pulse yields the focused field

(14) $$\begin{array}{ll} X_{1}\left(x,t^{\prime }\right) & =\sqrt{\frac{q}{q_{1}}}e^{\frac{ik_{0}x^{2}}{2q_{1}}}e^{-\frac{t^{\prime 2}}{t_{0}^{2}}}{\left(1-\frac{iqz}{q_{1}z_{R}}\right)}^{\frac{n}{2}}\\ & \;\times \frac{1}{2^{n}}H_{n}\left[\sqrt{\frac{1}{1-\frac{iqz}{q_{1}z_{R}}}}\left(\frac{t^{\prime }}{t_{0}}\pm \frac{x}{x_{0}}\frac{q}{q_{1}}\right)\right], \end{array}$$

where 1/*q* = −1/*f* + 1/*q*_0_ and *q*_1_ = *q* + *z* as above, which was obtained in [21] but is repeated here for the detailed SS analysis in next section. The focused field is a diverging, elliptical STOV at the focal plane of TC ±*n*, as appreciated in Figure 2(c), the transverse OAM of which is imparted by the lens [21]. As seen in Figure 2(d), a Fourier transforming lens produces the same STOV but without the diverging wave front, i.e.,

(15) $$X_{1}\left(x,t^{\prime }\right)=\sqrt{\frac{-ix_{0}}{x_{0,f}}}e^{-\frac{x^{2}}{x_{0,f}^{2}}}e^{-\frac{t^{\prime 2}}{t_{0}^{2}}}{\left(\frac{t^{\prime }}{t_{0}}\mp i\frac{x}{x_{0,f}}\right)}^{n},$$

where *x*_0,*f*_ = 2*f*/*k*_0_ *x*_0_ as above, with transverse OAM also imparted by the lens, and an *imaging system* reproduces a ST THL pulse,

(16) $$X_{1}\left(x,t^{\prime }\right)=\sqrt{\frac{1}{M}}e^{\frac{ik_{0}x^{2}}{R_{1}}}e^{-\frac{x^{2}}{x_{1}^{2}}}e^{-\frac{t^{\prime 2}}{t_{0}^{2}}}\frac{1}{2^{n}}H_{n}\left(\frac{t^{\prime }}{t_{0}}\pm \mathrm{sgn}\left(M\right)\frac{x}{x_{1}}\right),$$

with *x*_1_ = |*M*|*x*_0_, *R*_1_ = *M*/*C*, and opposite tilt, as in Figure 2(e), if *M* < 0. In the case of the 4*f* system, the transverse OAM supplied by the lens is eliminated by the second one.

## 3 Spectrospatial analysis

From their discovery [3], most of the theoretical analysis and experiments with STOVs/ST THL pulses are performed in the direct ST domain [4], [5], [6], [7], [8], also in the experiments of second harmonic [10], [11] and third harmonic [13] generation. Exceptions are those involving extremely short wave lengths in the extreme ultraviolet and soft X-rays range [14], [15], [16], given the current limitations in the direct ST characterization at these wave lengths, and where a SS characterization is the standard one. STOVs at these wave lengths are in fact being identified from the observation of numerical and experimental spatiospectra. It is then surprising that the spatiospectra of STOVs have only been evaluated analytically for unit TC [14]. A precise knowledge of the SS structure of STOVs/ST THL pulses provided by closed-form, simple expressions is fundamental for understanding and interpreting these and future experiments.

It turns out that the temporal frequency spectrum $\hat{X}\left(x,\Omega \right)=\left(1/2\pi \right)\int_{-\infty }^{\infty }dt^{\prime }X\left(x,t^{\prime }\right){\text{e}}^{\text{i}\Omega t^{\prime }}$ of the STOV in Eq. (3) is the SS THL pulse

(17) $${\hat{X}}_{0}\left(x,\Omega \right)=\frac{1}{2^{n}}e^{-\frac{\Omega^{2}}{\Omega_{0}^{2}}}e^{-\frac{x^{2}}{x_{0}^{2}}}H_{n}\left(\frac{\Omega }{\Omega_{0}}\pm \frac{x}{x_{0}}\right),$$

where Ω = *ω* − *ω*_0_ is the detuning from the carrier frequency *ω*_0_ and Ω_0_ = 2/*t*_0_ is the (narrow) spectral width. An irrelevant factor ${\left(-i\right)}^{n}/\sqrt{\pi }\Omega_{0}$ is omitted in Eq. (17). Similarly, the temporal frequency spectrum of the ST THL pulse in Eq. (11) is the SSOV

(18) $${\hat{X}}_{0}\left(x,\Omega \right)=e^{-\frac{\Omega^{2}}{\Omega_{0}^{2}}}e^{-\frac{x^{2}}{x_{0}^{2}}}{\left(\frac{\Omega }{\Omega_{0}}\mp i\frac{x}{x_{0}}\right)}^{n},$$

where $i^{n}/\sqrt{\pi }\Omega_{0}$ is omitted. Actually, the fact that STOVs and ST THL pulses are not only spatial Fourier transform pairs but also temporal Fourier transform pairs is an obvious fact from the symmetric role of *x* and *t*′ in their expressions, which however has not been noticed. The two standard outputs pulses from a 4*f* pulse shaper when a tilted *π*-step phase plate or a spiral phase plate are placed at the common focus are then doubly related by a spatial Fourier transform and a temporal Fourier transform.

We can then Fourier transform in time the generalized Fresnel diffraction integral in Eq. (2) to write

(19) $${\hat{X}}_{1}\left(x,\Omega \right)=\sqrt{\frac{k_{0}}{2i\pi B}}\int dx^{\prime }{\hat{X}}_{0}\left(x^{\prime },\Omega \right)e^{\frac{ik_{0}}{2B}\left(Dx^{2}-2xx^{\prime }+Ax^{\prime 2}\right)},$$

which is formally the same as its ST counterpart in Eq. (2) On doing this, we are ignoring the slight differences of propagation with frequency, which is justified and the customary approach for the narrow-band, many-cycle pulses considered in this paper, and is ultimately validated by the numerical and experimental observations. The propagated spatiospectrum of the STOV can then be obtained from the propagated ST THL pulse by simply making the replacement *t*′/*t*_0_ → Ω/Ω_0_ in its expression (12):

(20) $$\begin{array}{ll} {\hat{X}}_{1}\left(x,\Omega \right) & =\sqrt{\frac{1}{A\;+\;\;B/q_{0}}}e^{\frac{ik_{0}x^{2}}{2q_{1}}}e^{-\frac{\Omega^{2}}{\Omega_{0}^{2}}}{\left(\frac{A}{A\;+\;\;B/q_{0}}\right)}^{\frac{n}{2}}\\ & \;\times \frac{1}{2^{n}}H_{n}\;\left[\;\sqrt{\frac{A\;+\;\;B/q_{0}}{A}}\;\left(\frac{\Omega }{\Omega_{0}}\pm \frac{x}{x_{0}}\frac{1}{A\;+\;\;B/q_{0}}\right)\;\right]. \end{array}$$

Analogously, the propagated spatiospectrum of the ST THL pulse is obtained from the same replacement in the expression (4) of the propagated STOV:

(21) $$\begin{array}{ll} {\hat{X}}_{1}\left(x,\Omega \right) & =\sqrt{\frac{1}{A\;+\;\;B/q_{0}}}e^{\frac{ik_{0}x^{2}}{2q_{1}}}e^{-\frac{\Omega^{2}}{\Omega_{0}^{2}}}{\left(\frac{B/q_{0}}{A\;+\;\;B/q_{0}}\right)}^{\frac{n}{2}}\\ & \;\times \frac{1}{2^{n}}H_{n}\;\left[\;\sqrt{\frac{A\;+\;\;B/q_{0}}{B/q_{0}}}\;\left(\frac{\Omega }{\Omega_{0}}\mp i\frac{x}{x_{0}}\frac{1}{A\;+\;\;B/q_{0}}\right)\;\right]. \end{array}$$

Thus, the spatiospectrum of the STOV is identical and propagates identically to the ST THL pulse, and as such is plotted at the output plane of the various optical systems in the same Figure 2 as the ST THL pulse and indicated by SS THL pulse. Vice versa, the spatiospectrum of the ST THL pulse behaves identically as a STOV, and is plotted at the exit of the optical systems in the same Figure 1 as the STOV with the name SSOV.

**Figure 3:**
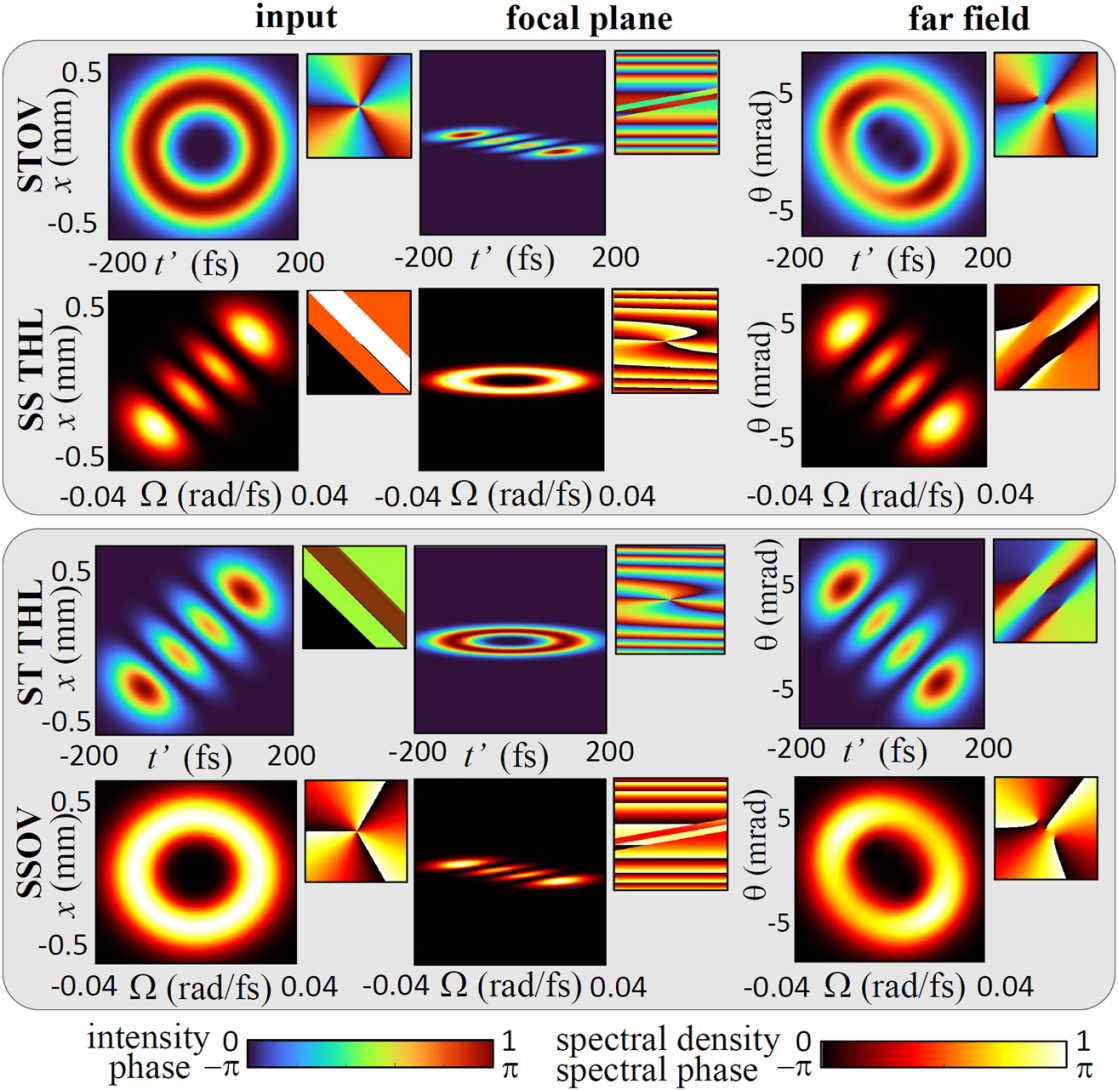
Focusing and propagation up to the far field of STOVs and ST THL pulses of TC +3, observed spatiotemporally (odd rows) and spatiospectrally (even rows). In this example *ω*_0_ = 2.8 rad/fs, *x*_0_ = 0.3 mm, *t*_0_ = 100 fs, and *f* = 75 mm.

Let us illustrate this double duality with focusing the output of a STOV-THL-forming 4*f* pulse shaper, which is also the standard setup in experiments of high harmonic and attosecond pulse generation, including here the propagation up to the far field as the observation region. Equations (7) and (14) are then modified to their limiting far-field forms as functions of the observation angle *θ* = *x*/*z*:

(22) $$X_{\text{far}}\left(\theta ,t^{\prime }\right)=e^{-\frac{\theta^{2}}{\theta_{1}^{2}}}e^{-\frac{t^{\prime 2}}{t_{0}^{2}}}{\left(\frac{iq}{z_{R}}\right)}^{\frac{n}{2}}\frac{1}{2^{n}}H_{n}\left[\sqrt{\frac{z_{R}}{iq}}\left(\frac{t^{\prime }}{t_{0}}\mp i\theta \frac{q}{x_{0}}\right)\right]$$

for the focused STOV far field, and

(23) $$\begin{array}{ll} X_{\text{far}}\left(\theta ,t^{\prime }\right) & =e^{-\frac{\theta^{2}}{\theta_{1}^{2}}}e^{-\frac{t^{\prime 2}}{t_{0}^{2}}}{\left(1-\frac{iq}{z_{R}}\right)}^{\frac{n}{2}}\\ & \;\times \frac{1}{2^{n}}H_{n}\left[\sqrt{\frac{1}{1-\frac{iq}{z_{R}}}}\left(\frac{t^{\prime }}{t_{0}}\pm \theta \frac{q}{x_{0}}\right)\right] \end{array}$$

for the focused ST THL pulse. The decay and curvature factors $\sqrt{q/z}\;e^{ik_{0}x^{2}/2z}$ are omitted, and the divergence angle is given by

(24) $$\theta_{1}=\frac{x_{0}}{f}\sqrt{1+\frac{f^{2}}{z_{R}^{2}}},$$

which can be approximated by *θ*_1_ ≃ *x*_0_/*f* for strong focusing (meaning negligible focal shift but still paraxial). Equations (7), (14), (22) and (23) provide for the first time the complete picture of propagating STOV-THL pulses in standard experiments as would be observed with field characterization techniques with spatiotemporal resolution. In Figure 3 we have deliberately chosen a soft focusing geometry to enhance the intricate far field structure of STOVs (first row), where the vortices never merge in a perfectly elliptical STOV, as it would contradict the conservation of the transverse OAM in free space propagation. The transverse OAM of the focused elliptical STOV is ±*nγ*/2*ω*_0_, whereas an elliptical STOV at the far field would have ∓*nγ*_far_/2*ω*_0_ → 0 as *γ*_far_ = *ct*_0_/*x*_far_ → 0 given the linearly increasing width *x*_far_ at the far field. Similarly the far field structure of the focused ST THL pulse (third row) is never another ST THL pulse with *n π*-steps in the phase, but the vortices remain up to infinite propagation distances even if strongly deformed.

The second and four rows of Figure 3 illustrate the SS picture as detected by a spectrometer with spatial resolution. The complete picture is obtained in analytical terms by replacing *t*′/*t*_0_ with Ω/Ω_0_ in Eq. (7) for STOV focusing and in Eq. (14) for ST THL focusing, and interchanging the equations. The same for Eqs. (22) and (23). The result is

(25) $$\begin{array}{ll} {\hat{X}}_{1}\left(x,\Omega \right) & =\sqrt{\frac{q}{q_{1}}}e^{\frac{ik_{0}x^{2}}{2q_{1}}}e^{-\frac{\Omega^{2}}{\Omega_{0}^{2}}}{\left(1-\frac{iqz}{q_{1}z_{R}}\right)}^{\frac{n}{2}}\\ & \;\times \frac{1}{2^{n}}H_{n}\left[\sqrt{\frac{1}{1-\frac{iqz}{q_{1}z_{R}}}}\left(\frac{\Omega }{\Omega_{0}}\pm \frac{x}{x_{0}}\frac{q}{q_{1}}\right)\right], \end{array}$$

for the SS THL pulse of the focused STOV, and

(26) $$\begin{array}{ll} {\hat{X}}_{1}\left(x,\Omega \right) & =\sqrt{\frac{q}{q_{1}}}e^{\frac{ik_{0}x^{2}}{2q_{1}}}e^{-\frac{\Omega^{2}}{\Omega_{0}^{2}}}{\left(\frac{iqz}{q_{1}z_{R}}\right)}^{\frac{n}{2}}\\ & \;\times \frac{1}{2^{n}}H_{n}\left[\sqrt{\frac{q_{1}z_{R}}{iqz}}\left(\frac{\Omega }{\Omega_{0}}\mp i\frac{x}{x_{0}}\frac{q}{q_{1}}\right)\right], \end{array}$$

for the SSOV of the focused ST THL pulse, with respective far fields

(27) $$\begin{array}{ll} {\hat{X}}_{\text{far}}\left(\theta ,\Omega \right) & =e^{-\frac{\theta^{2}}{\theta_{1}^{2}}}e^{-\frac{\Omega^{2}}{\Omega_{0}^{2}}}{\left(1-\frac{iq}{z_{R}}\right)}^{\frac{n}{2}}\\ & \;\times \frac{1}{2^{n}}H_{n}\left[\sqrt{\frac{1}{1-\frac{iq}{z_{R}}}}\left(\frac{\Omega }{\Omega_{0}}\pm \theta \frac{q}{x_{0}}\right)\right] \end{array}$$

for the SS THL pulse, and

(28) $$\begin{array}{ll} {\hat{X}}_{\text{far}}\left(\theta ,\Omega \right) & =e^{-\frac{\theta^{2}}{\theta_{1}^{2}}}e^{-\frac{\Omega^{2}}{\Omega_{0}^{2}}}{\left(\frac{iq}{z_{R}}\right)}^{\frac{n}{2}}\\ & \;\times \frac{1}{2^{n}}H_{n}\left[\sqrt{\frac{z_{R}}{iq}}\left(\frac{\Omega }{\Omega_{0}}\mp i\theta \frac{q}{x_{0}}\right)\right] \end{array}$$

for the SSOV. As evidenced in Figure 3, focusing of the STOV viewed spatiospectrally is identical to focusing the ST THL pulse in space-time, and focusing of the ST THL viewed spatiospectrally is identical to focusing of the STOV in space-time.

## 4 Conclusions

Although spatiotemporal optical vortices are not modes of propagation, the spatiotemporal/spatiospectral duality in which vortices appear in the spatial spectrum in the form of spatiospectral vortices when they disappear in space-time, confers them with a sophisticated unity that had not previously been noticed. Once the problem of the transverse orbital angular momentum has been clarified [23], the present theory provides a full understanding from closed-form analytical expressions of the transformation properties of STOVs of arbitrary topological charge through rather general optical systems as *ABCD* systems. The formulation in spatiospectral domain as spatiospectral tilted Hermite-lobed pulses and spatiospectral optical vortices is particularly relevant in experiments where STOVs are created in the extreme ultraviolet and soft X-ray wave length regimes, where the standard characterization techniques are substantially based on the observation of spatiospectra. Overall, the precise knowledge of the spatiotemporal and spatiospectral structure of STOVs provided here will lead to decisive advances in the design and precision of their applications. Further theoretical and experimental efforts are necessary to describe and generate broadband, few-cycle STOVs propagating non-paraxially, as the electromagnetic STOVs reported in Ref. [29].
